# Genomics and Systems Biology at the “Century of Human Population Genetics” conference

**DOI:** 10.1186/s12864-020-06993-1

**Published:** 2020-09-10

**Authors:** Tatiana V. Tatarinova, Ancha V. Baranova, Anastasia A. Anashkina, Yuriy L. Orlov

**Affiliations:** 1 La Verne University, La Verne, CA 91750 USA; 2 Department of Fundamental Biology and Biotechnology, Siberian Federal University, 660074 Krasnoyarsk, Russia; 3 George Mason University, Fairfax, VA 22030 USA; 4 Research Centre for Medical Genetics, 115522 Moscow, Russia; 5 Engelhardt Institute of Molecular Biology RAS, 119991 Moscow, Russia; 6 The Digital Health Institute, I.M.Sechenov First Moscow State Medical University (Sechenov University), 119991 Moscow, Russia; 7 Institute of Cytology and Genetics SB RAS, 630090 Novosibirsk, Russia; 8 Novosibirsk State University, 630090 Novosibirsk, Russia

This special issue of BMC Genomics presents works in the broad field of genomics as discussed at the “Century of Human Population Genetics” conference in Moscow in May 2019, held at Moscow State University (http://centenary-popgene.com/). The Conference discussed the current research of gene pools of the world’s nations, analysis of ancient DNA, judicial possibilities of human genetics, developemngt of population genetic databases, biobanks, as well as a set of newest additions to the toolbox of genomics technologies. It was an unique event, dedicated to the 100th anniversary of the first human population study performed in 1919, way before a function of DNA was discovered.

This journal issue also contains materials on human genomics and computational genetics presented at the “Systems Biology and Bioinformatics” (SBB-2019), a School for the Young Scientists held in Novosibirsk, Russia. Since 2008, this traditional school on bioinformatics is organized annually, under joint guidance of the Institute of Cytology and Genetics of the Siberian Branch of the Russian Academy of Sciences and the Novosibirsk State University (http://conf.bionet.nsc.ru/sbb2019/en/) [1, 2]. Traditionally, the Program Committee selects best conference materials for subsequent publications in BMC Genomics and related journals of BioMed Central [3–6]. The SBB Schools in Novosibirsk are the satellite events for the larger BGRS\SB (Bioinformatics of Genome Regulation and Structure \ Systems Biology) multiconference [1]. At the time of this paper writing, the BGRS\SB-2020 meeting in Novosibirsk just concluded (https://bgrssb.icgbio.ru/2020/), for the first time its twenty years history in the on-line format.

Therefore, this special issue on genomics and systems biology is accompanied by sister issues in other BMC journals in the fields of genetics, bioinformatics, microbiology, and medical genomics are published as a part of the following series: BMC Bioinformatics, BMC Medical Genomics, BMC Genetics, and BMC Medical Genetics, as well as in BMC Microbiology. In 2018, the conference highlights were organized into the Special Issues with reports from the BGRS\SB-2018 Conference and, earlier, from Belyaev Readings-2017 in Novosibirsk [7, 8]. The special issues in BMC Genomics were continued in 2019 [9, 10].

We open up this Special Issue by the human population genomics study on genetic determinants for the eye and hair color by Balanovska et al. [11] (This issue).

Predicting the eye and hair color from genotype became an established and widely used tool for both the forensic genetics and for a studies of ancient human populations. Accuracy of these predictions were extensively verified in the West and Central Europeans [12], while the studies in lightly pigmented people from border regions between Europe and Asia, including Caucasus and Ural are lacking. The authors collected 300 samples from across northern Eurasia, phenotyped and genotyped them using HIrisPlex-S markers [13], then estimated the predictive power of these biomarkers in Caucasus/Ural/Siberian populations. As genetic ancestries of these populations differ from that of West Europeans, Balanovska and co-authors hypothesized that they allelic spectrum might be also somewhat different. Thus, for all the 300 samples, the authors performed the exome sequencing and performed an enrichment of biomarker set with the 53 genes and intergenic regions associated with the eye/hair color. The association analysis replicated previous finsings concerning some known pigmentation related SNPs but also identified five new markers, with eye color prediction power for North Eurasians comparable with that of two well-known SNPs of this type. Four of these SNPs are located in *HERС2* gene. The released dataset may be used for further advancement of population genetics and medical genetics; it describes the exome variation in some undercharacterized indigenous groups, previously studied by SNPs arrays only, but not by the sequencing approach.

The paper by Sandoval and colleagues [14] (this issue) continues topic of human population genetics with an excurse to Latin American and a journey of ‘Canaris’ people from Ecuador and Peru. According to pre-Hispanic history records, during the conquest and Inka expansion in Ecuador, many Andean families of the Cañar region were been displaced to several places, including Kañaris, a Quechua-speaking community of Peru mountains. The study focused on the genetic footprints of the ‘Cañaris’ of Cañar compared to other highland populations. The authors analyzed native Y chromosome haplotypes of local communities—three from Ecuador and seven from Peru – to show that individuals from the Cañar region do not share Y haplotypes with the Kañaris. Although no close genetic links between the Peruvian Kañaris (including Inkawasi) and Ecuadorian Cañar populations were shown, some congruence with historical records was observed [15].

Suntsova and Buzdin [16] (this issue) review compares human and great apes genomes to reveal genetic features distinguishing us from chimpanzees and making us humans. Even if these features are quantifiable, we still cannot identify with certainty the causative genes of “human identity” [17]. The authors summarize available information about genetic differences between humans and chimpanzees and potential functional impacts of these on differences on molecular, anatomical, physiological, and cognitive features of these species.

The paper by Kirill Danilov and colleagues [18] (this issue) investigates a performance of commonly used genotyping technologies, including Whole Genome (WGS) and Whole Exome Sequencing (WES) [13]. They conclude that WGS genotype callings exhibit higher overall precision within the selected variety of discordantly genotyped variants, a finding relevant to clinical diagnostics of common and rare variants.

The work by Vasilina Akulova et al. [19] (this issue) focuses on a basidiomycete tree fungus (*Armillaria borealis)* genome. This fungus causes the “white rot” root disease that weakens and even kills woody plants; it is common in Siberia and the Far East [20]. The de novo genome assembly and annotation were performed for the *A. borealis* species for the first time. Functional annotation analysis revealed about 22,000 protein-coding genes and provided data for further comparative analysis with other fungal species. Note that the work by Akulova et al. (this issue) refers to to BMC Plant Biol special issues published in parallel after PlantGen-2019 Conference (http://conf.bionet.nsc.ru/plantgen2019/en/), which continues as a series of special issues at BMC family journals [21].

Through offering schools for young scientists, we aim to support international exchanges and education in the field of computational biology and bioinformatics (https://bgrssb.icgbio.ru/2020/). We invite our worldwide readers to attend our next events – in Novosibirsk and Moscow (https://forum.digital/clinic, http://ngs.med-gen.ru/).
